# Identification of functional SNP associated with sperm quality in porcine *ANXA5* that contributes to the growth of immature Sertoli cell

**DOI:** 10.3389/fvets.2025.1576566

**Published:** 2025-05-14

**Authors:** Mingyang Han, Diwen Yao, Yuyang Song, Yuhang Liu, Zhihua Chen, Jialian Li, Fenge Li, Xiuqin Yang, Lihe Dai, Buyue Niu

**Affiliations:** ^1^College of Animal Science and Technology, Northeast Agricultural University, Harbin, China; ^2^Key Laboratory of Pig Genetics and Breeding of Ministry of Agriculture & Key Laboratory of Agricultural Animal Genetics, Breeding and Reproduction of Ministry of Education, Huazhong Agricultural University, Wuhan, China; ^3^Zhejiang Key Laboratory of Livestock and Poultry Biotech Breeding, Institute of Animal Husbandry and Veterinary Science, Zhejiang Academy of Agricultural Sciences, Hangzhou, China

**Keywords:** Anxa5, ESR1, boar, semen traits, Sertoli cell

## Abstract

AnnexinA5 (ANXA5) has been identified as a positional candidate gene for reproduction and fertility traits in boars, but its role in testicular tissue development, as well as genetic variations remain unclear. The aim of this study was to explore the effect of ANXA5 in the growth of swine Sertoli cells and identify its functional variations. Firstly, the expression of porcine ANXA5 in different tissues was detected by semi-quantitative RT-PCR and its effect on the proliferation of Sertoli cells was evaluated by CCK8, EdU, flow cytometry analyses and qRT-PCR. Then, putative causative variants were screened by integrating in silico analysis and DNA sequencing, and the subsequent association analysis was performed in Largewhite boars. Lastly, dual luciferase reporter assay was used to clarify the effect of specific SNP or ESR1 on ANXA5 transcription. The results showed that ANXA5 expressed in all the detected tissues, promoted proliferation of Sertoli cells by advancing cell cycle progression from the G1 to S phase and encouraging expression of PCNA. Putative causative variants, including two ns-SNPs within the coding region, and three closely linked SNPs in the promoter region were identified. Statistical analysis showed that the frequency of the T allele at g.-676 T > C, A allele at g.-674C > A, and T allele at g.-105G > T were each 0.75, the heterozygotes of Yorkshire boars had greater sperm motility as compared to TT, AA, and TT animals (*p* < 0.05). Luciferase reporter analysis suggested g.-105G > T and ESR1 modulated ANXA5 transcription. Taken together, this study demonstrated ANXA5 affected swine immature Sertoli cells growth and g.-105G > T was a candidate genetic marker for reproductive trait of boar.

## 1 Introduction

Artificial insemination with boar semen is widely used in modern pig production. Semen quality and quantity are moderately heritable, indicating the possibility to improve sperm traits through breeding (1, 2). Genetic selection of sperm traits involves identifying causative genes or DNA variants. Genome-wide association studies (GWAS) are widely used to identify single nucleotide polymorphisms (SNPs) in linkage disequilibrium associated with quantitative trait loci and candidate genes. Six significant SNPs associated with boar sperm motility were identified by GWAS (3), including mitochondrial methionyl-tRNA formyltransferase as a candidate gene associated with semen traits of the Yorkshire boar. Weighted single-step GWAS has been applied to identify quantitative trait loci associated with sperm motility, as well as other sperm quality and quantity traits in Yorkshire (4), Duroc (5–7), and Piétrain boars (8). By integrating GWAS and RNA sequencing, Gòdia et al. (9) generated a gene interaction network and identified candidate genes, pathways, and DNA variants associated with sperm quality. Although various markers and candidate genes have been identified by GWAS, these findings are insufficient to explain the molecular basis of sperm quality and limited identification of causative genes and genetic variants.

AnnexinA5 (ANXA5) is a member of the Annexin A family of structurally related proteins that bind to phospholipids in a calcium-dependent manner (10, 11). ANXA5 is a placental anticoagulant protein that plays a role in blood supply to the fetus throughout gestation. Genetic variants of ANXA5, especially the promoter H2 haplotype, have been associated with an increased risk of recurrent pregnancy loss (12). In humans and rodents, ANXA5 is expressed in the testis (13), epididymis (14), sperm (15), pituitary endocrine cells, testicular Leydig cells, and Sertoli cells (12, 16). However, the influence of *ANXA5* genetic variants on male fertility remains unclear (10).

Porcine *ANXA5* has been identified using GWAS as a potential candidate gene associated with reproductive and fertility traits of Yorkshire boars (17). Sperm quality is crucial for successful fertilization, litter size, and piglet mortality, which contribute to the profitability of modern pig production. Sperm morphology, motility, and fertilization capacity are determined by multiple genetic and environmental factors. The efficiency of spermatogenesis partially depends on the number of Sertoli cells per testis, which is limited by the proliferation rate (18, 19). However, the biological role of porcine *ANXA5* in Sertoli cell growth remains unknown.

Therefore, the present study aimed to characterize porcine *ANXA5*, clarify its role in the growth of swine immature Sertoli cells and to identify genetic variations associated with boar semen traits, thereby providing novel markers to enhance the quantity of boar semen.

## 2 Materials and methods

### 2.1 Animals and tissue collection

As described in a previous report (20), the heart, liver, spleen, lung, kidney, stomach, duodenum, jejunum, ileum, cecum, colon, rectum, and testis were collected from four specific pathogen-free piglets (age, 28 days; Harbin Veterinary Research Institute, Harbin, China). Total RNA was extracted and reverse transcribed into complementary DNA (cDNA), which was stored at −20°C. Genomic DNA samples of 10 Min and 10 Landrace pigs stored in our laboratory were used to identify genetic variants of the porcine *ANXA5* gene (20).

For the association analysis, sperm samples were collected from 417 randomly selected Yorkshire boars (Guangxi Yangxiang Co., Ltd., Guangxi, China). The semen traits included volume, concentration, motility, and abnormal spermatozoa rate. Genomic DNA was isolated using the phenol and chloroform extraction method as described by Zhao et al. (21).

### 2.2 Primer design, polymerase chain reaction (PCR), and reverse transcription-PCR (RT-PCR)

The primer pairs used in this study were designed using Primer 5 software (Premier Biosoft, San Francisco, CA, USA) based on the mRNA sequences of porcine *ANXA5* (XM_003129218.5), *ESR1* (ENSSSCT00000035147.2), and *PCNA* (NM_001291925). The primer sequences are presented in Supplementary Table S1.

Each reaction volume (20 μL) for PCR and semi-quantitative RT-PCR analyses consisted of DNA or cDNA templates (100–150 ng), 2 × Taq Master Mix (10 μL; Takara Biotechnology Co., Ltd., Dalian, China), and appropriate gene-specific primers (0.5 μM). The amplification conditions consisted of an initial denaturation step at 94°C for 4 min, followed by 35 or 29 cycles of denaturation at 94°C for 30 s, annealing at the annealing temperature shown in Table S1 for 30 s, extension at 72°C for 1 min, and a final extension step at 72°C for 10 min.

Each reaction volume (20 μL) for qRT-PCR analysis consisted of a cDNA template (100 ng), SYBR master mix (10 μL; Takara Biotechnology Co., Ltd.), and gene-specific primers (0.2 μM). Amplification was performed using an ABI QuantStudio 3 Real-Time PCR System (Applied Biosystems, Foster City, CA, USA). The amplification conditions consisted of an initial denaturation step at 95°C for 30 s, followed by 40 cycles of denaturation at 95°C for 5 s and extension at 60–62°C for 35 s. Melting curves were generated. Relative mRNA expression of specific genes in ST cells was calculated with the 2^-ΔΔCT^ method against glyceraldehyde-3-phosphate dehydrogenase (*GAPDH*) as an internal reference gene (22).

### 2.3 *In silico* analysis

Protein sequences of ANXA5 were retrieved from the NCBI database (https://www.ncbi.nlm.nih.gov/), which included *Homo sapiens* (NM_001154.4), *Pan troglodytes* (NM_001009099.1), *Macaca mulatta* (XM_015139155.2), *Equus caballus* (XM_023636621.1), *Canis lupus familiaris* (NM_001314118.1), *Sus scrofa* (XM_003129218.5), *Bos taurus* (NM_001040477.4), *Ovis aries* (XM_012179394.4), *Mus musculus* (NM_009673.2), *Rattus norvegicus* (NM_013132.2), *Gallus gallus* (NM_001031538.2), *Xenopus tropicalis* (NM_001008182.1), and *Gopherus evgoodei* (XM_030564272.1). Alignment of ANXA5 sequences from different species or *ANXA5* genomic sequences from different pigs was performed using DNAMAN software (https://www.lynnon.com/). A phylogenetic tree of the ANXA5 sequences was generated with Molecular Evolutionary Genetics Analysis 7 software (https://www.megasoftware.net/) with the neighbor-joining method. Proteins that might interact with porcine or human ANXA5 were retrieved from the STRING database (https://string-db.org/). Functional non-synonymous SNPs (ns-SNPs) of the porcine *ANXA5* gene were predicted using the Polymorphism Phenotyping v2 tool (http://genetics.bwh.harvard.edu/pph2/), SNAP software (https://www.broadinstitute.org/snap/snap), SIFT (https://useast.ensembl.org/Sus_scrofa/Transcript/Variation_Transcript/Table?db=core;g=ENSSSCG00000009097;r=8:102388103-102425750;t=ENSSSCT00000009963), and PhD-SNP (https://snps.biofold.org/phd-snp/phd-snp.html).

The core region of the *ANXA5* promoter was predicted with the Promoter 2.0 database (https://services.healthtech.dtu.dk/services/Promoter-2.0/) and the neural network promoter prediction database (https://www.fruitfly.org/seq_tools/promoter.html). Potential transcription factor binding sites were predicted using the JASPAR database (http://jaspar.binf.ku.dk/cgi-bin/jaspar_db.pl).

### 2.4 Overexpression vectors and small interfering RNA (siRNA)

For construction of overexpression vectors, the coding region of porcine *ANXA5* or *ESR1* was amplified with gene-specific primers (Table S1), cloned between the *Kpn*I and *Xho*I or *Eco*RI restriction sites of the pcDNA3.1 or pCMV-HA expression vector, verified by restriction endonuclease, and named as pcDNA3.1-ANXA5 or pCMV-HA-ESR1, respectively. As shown in Table S1, *ANXA5*-siRNA and a negative control (NC) were designed and commercially synthesized(General boil, Chuzhou, China).

To explore the function of the *ANXA5* promoter, four luciferase reporter gene vectors were constructed. The putative promoter region of porcine *ANXA5* was amplified using primer pairs ANXA5-P (Table S1), cloned between the *Kpn* I and *Xho* I restriction sites of the pGL3-Basic luciferase reporter gene vector (Promega Corporation, Madison, WI, USA), and named pGL3-ANXA5. With pGL3-ANXA5 as a template, four 5′ deletion fragments of the *ANXA5* promoter were amplified with the primer pairs ANXA5-J1 to ANXA5-J4 (Table S1) and inserted into the pGL3-Basic vector (Promega Corporation), which were named pGL3-ANXA5-J1 to J4, respectively. To evaluate the effect of g.-105G > T, a mutant termed ANXA5-J4 (TT) with a putative ESR1 binding site, was amplified with mutagenic primers (Table S1), cloned into the pGL3-Basic vector, and named pGL3-ANXA5-TT. All constructed vectors were double-confirmed by DNA sequencing performed by Sangon Biotech (Shanghai) Co., Ltd. (Shanghai, China).

### 2.5 Cell transfection, cell proliferation assay, and cell cycle assay

ST cells (American Type Culture Collection, Manassas, VA, USA) were cultured in high-glucose Dulbecco’s modified Eagle’s medium supplemented with 10% fetal bovine serum (Gibco, Carlsbad, CA, USA) at 37°C under an atmosphere of 5% CO_2_/95% air, and transfected with 0.1 μg of pcDNA3.1-ANXA5 (*ANXA5*-siRNA) or pcDNA3.1 (siRNA NC) using 0.3 μL of X-treme GENE HP DNA Transfection Reagent (Roche Diagnostics GmbH, Mannheim, Germany). After 48 h, the cells were collected for the cell proliferation and cell cycle assays.

In line with the protocol of Cell Counting Kit-8 (CCK-8) (Beijing Seven Dimension Biotechnology, Co., Ltd., Beijing, China), ST cells were plated in the wells of 96-well plates with 10 μL of CCK-8 solution at 37°C for 2 h. Afterward, the optical density at a wavelength of 450 nm was detected using a microplate reader (Infinite F50; Tecan Group AG, Männedorf, Switzerland). Cell viability was calculated as [A (spiked) - A (blank)] / [A (0 spiked) - A (blank)] × 100. In accordance with the manufacturer’s instructions for the EdU (5-ethynyl-2′-deoxyuridine) Cell Proliferation Kit with Alexa Fluor 555 (Beyotime Biotech Inc., Shanghai, China), ST cells were fixed in EdU solution, stained with Azide 555 (red) and Hoechst 33342 (blue), and imaged under a fluorescence inverted microscope (Leica Microsystems GmbH, Wetzlar, Germany). The proportion of EdU-positive cells was determined using ImageJ software (https://imagej.net/ij/). In accordance with the manufacturer’s instructions of the cell cycle staining kit (MultiSciences Biotech Co., Ltd., Hangzhou, China), ST cells were cultured in the wells of six-well plates, mixed with 1 mL of DNA staining solution and 10 μL of permeabilization solution, and incubated for 30 min at room temperature. The proportions of cells in different phases of the cell cycle were quantified using a FACSCelesta flow cytometer (FACSCelesta; BD Biosciences, San Diego, CA, USA).

### 2.6 Dual luciferase reporter assays

To identify the core region of the *ANXA5* promoter, ST cells were transiently transfected with luciferase reporter gene vectors (0.5 μg) or a negative control (0.005 μg; pGL3-basic) and of internal control (pRL-TK) with 1.5 μL of X-treme GENE HP DNA Transfection Reagent (Roche Diagnostics USA, Indianapolis, IN, USA). To validate the role of ESR1 on the transcriptional activity of *ANXA5*, ST cells were co-transfected with 0.25 μg of the luciferase reporter vector pGL3-ANXA5, pGL3-ANXA5-J4(GG) or pGL3-ANXA5-(TT), 0.25 μg of the overexpression plasmid pCMV-HA-ESR1 or pCMV-HA, and 0.005 μg of pRL-TK. According to the Dual-Luciferase Reporter Assay System (Promega Corporation), after transfection for 48 h, the cells were lysed, and the enzymatic activity of firefly and Renilla luciferase was assessed using an illuminometer Sirius L Illuminometer; Berthold Technologies GmbH & Co. KG, Bad Wildbad, Germany. Relative luciferase activity was calculated as the ratio of firefly to Renilla luciferase activity (Fluc/Rluc). All transfections were performed in triplicate and repeated three times.

### 2.7 Identification and genotyping of porcine ANXA5

*ANXA5* promoters were amplified from 10 Min pigs and 10 Landrace pigs using gene-specific primer pairs (Supplementary Table S1). Then, the fragments were purified and sequenced by Sangon Biotech (Shanghai) Co., Ltd. The obtained sequences were aligned using DNAMAN software and analyzed with the JASPER tool (https://github.com/alguoo314/JASPER). The fragments including three candidate functional SNPs from 417 Yorkshire pigs were amplified using gene-specific primer pairs (Table S1) and sequenced and genotyped with DNAMAN software.

### 2.8 Statistical analysis

Statistical analyses were performed using SPSS (IBM Corporation, Armonk, NY, USA). All data are presented as the mean ± standard error of the mean. Considering the continuity and regularity of gene expression, the present data were considered normally distributed. The differences in cell growth, gene expression, and transcriptional activity between groups were evaluated using the two-tailed Student’s *t*-test. The effects of SNPs on the semen traits were analyzed using the general linear model (GLM) procedure of SAS version 9.2.21 as follows:

*Y_ij_ = μ + G_i_ + F_j_ + e_ij_*, where *Y_ij_* is the observed trait, *μ* is the mean value of the trait, *G_i_* is the fixed effect with genotype, *F_j_* is the effect of the farm, and *e_ij_* is the random residual. A probability (*p*) value < 0.05 was considered statistically significant.

## 3 Results

### 3.1 Characterization of porcine *ANXA5*

**Figure 1:**
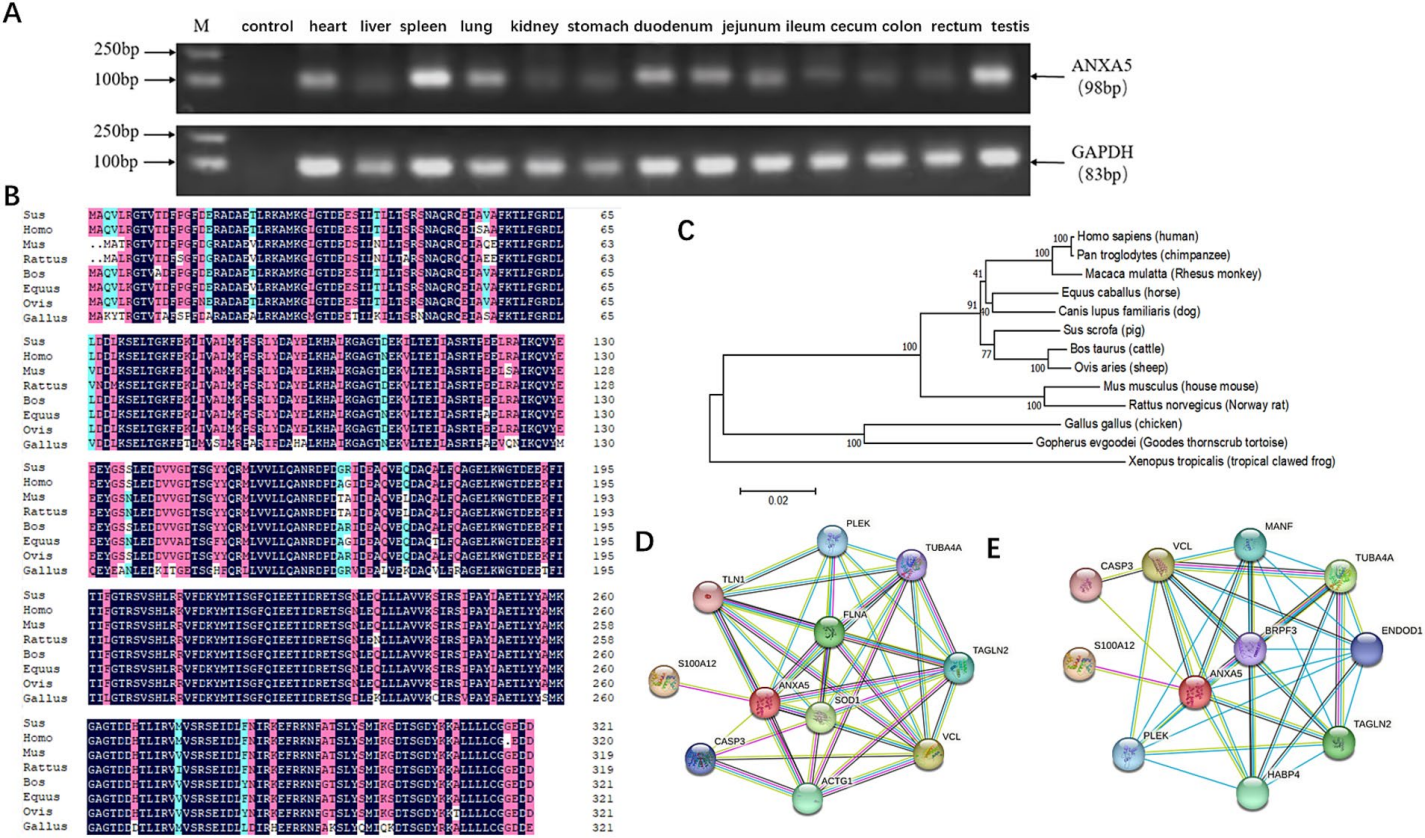
Characterization of porcine ANXA5. **(A)** Tissue expression profile of porcine ANXA5. **(B)** Multiple sequence alignment of ANXA5 protein in eight species. **(C)** Phylogenetic tree of ANXA5. **(D)** The predictive functional partner of human ANXA5. **(E)** The predictive functional partner of porcine ANXA5.

Semi-quantitative RT-PCR revealed that the porcine *ANXA5* gene was expressed in the heart, liver, spleen, lung, kidney, stomach, duodenum, jejunum, ileum, cecum, colon, rectum, and testis (Figure 1A). A 966-bp fragment of the coding sequence of porcine *ANXA5* was obtained by PCR amplification, which encoded 321 amino acids. Multiple protein sequence alignment confirmed that porcine *ANXA5* shares about 98% identity with humans, cattle, and sheep, and more than 90% identity with rats, mice, and horses (Figure 1B). Phylogenetic analysis showed that *ANXA5* of pigs, cattle, and sheep belong to the same cluster, while *ANXA5* of horses, dogs, and primates formed a separate cluster (Figure 1C). Protein–protein interaction network analysis revealed that proteins that interacted with *ANXA5* were highly conserved between pigs and humans. These proteins, including S100 calcium-binding protein A12 (S100A12), caspase-3 (CASP3), vinculin (VCL), pleckstrin (PLEK), tubulin alpha 4a (TUBA4A), and transgelin 2 (TAGLN2), participate in regulation of immune responses, cell proliferation, cell apoptosis, and cytoskeletal structure (Figures 1D,E).

### 3.2 *ANXA5* is involved in proliferation of immature Sertoli cells

**Figure 2:**
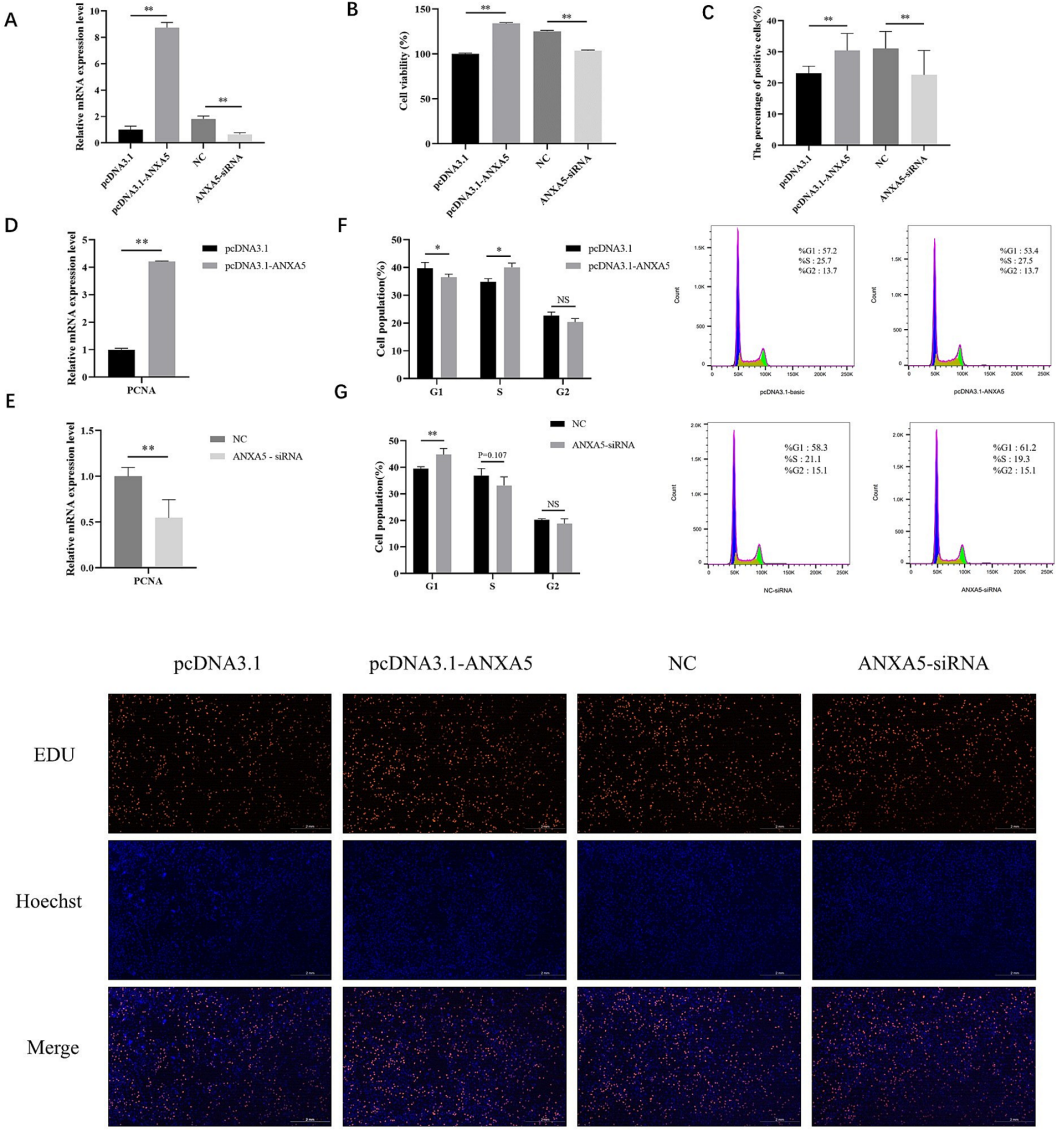
ANXA5 affects the proliferation of immature Sertoli cells. **(A)** The efficiency of porcine ANXA5 overexpression or interference. **(B)** Cell viability was detected by CCK8. **(C)** Cell proliferation was detected by EdU. Scale bar: 2 mm. **(D)** The effect of ANXA5 overexpression on the mRNA expression of *PCNA*. **(E)** The effect of ANXA5 interference on the mRNA expression of *PCNA*. **(F)** The effect of ANXA5 overexpression on the cell cycle. **(G)** The effect of ANXA5 interference on cell cycle. Values were shown as the mean ± SEM (*n* = 3). *p < 0.05; **p < 0.01.

To assess the role of *ANXA5* on the proliferation of immature Sertoli cells, ST cells were transfected with a porcine *ANXA5* overexpression vector or siRNA. As shown in Figure 2A, the results of qRT-PCR analysis showed the mRNA expression of *ANXA5* was significantly increased in ST cells transfected with the vector pcDNA3.1-ANXA5 (*p* < 0.01) and significantly decreased in ST cells transfected with ANXA5-siRNA (*p* < 0.01). The results of the CCK-8 assay showed that absorbance (450 nm) of *ANXA5*-overexpressed cells was significantly higher than the control group (*p* < 0.01), while significantly lower for *ANXA5*-siRNA transfected cells (*p* < 0.01) (Figure 2B). As compared to the corresponding control cells, the proportion of EdU-positive cells was significantly increased in the *ANXA5* overexpressed cells (*p* < 0.01) and significantly lower in *ANXA5*-siRNA transfected cells (*p* < 0.01) (Figure 2C). *PCNA* mRNA expression was significantly higher in pcDNA3.1-transfected cells (*p* < 0.01), while significantly decreased in *ANXA5*-siRNA-transfected cells (*p* < 0.01) (Figure 2D). The CCK-8 assay, EdU staining, and qRT-PCR analysis confirmed that *ANXA5* promoted proliferation of immature Sertoli cell by upregulating expression of *PCNA*.

Cell cycle analysis showed that the proportion of cells transfected with pcDNA3.1-ANXA5 was lower in the G1 phase (*p* < 0.05) and higher in the S phase (*p* < 0.05), as compared to the control (Figure 2F). Knockdown of *ANXA5* increased the proportion of cells in the G1 phase (*p* < 0.05) and decreased the proportion in the S phase (*p* = 0.107) (Figure 2G).

### 3.3 *In silico* analysis of ns-SNPs of porcine *ANXA5*

In the current study, 23 SNPs of the *ANXA5* coding region were retrieved from the Ensembl SNP database. Among these SNPs, nine ns-SNPs were identified and independently analyzed. As shown in Table 1, Polyphen-2, revealed that the polymorphism c.234G > C, c.286G > T, c.404G > A, and c.839C > T were damaging. SNAP showed that the polymorphism c.234G > C and c.286G > T were non-neutral and predicted by SIFT and PhD-SNP to both be deleterious. The polymorphism c.234G > C (R25P) and c.286G > T (L42F) were predicted to be possibly damaging, non-neutral, or deleterious and selected as candidate functional ns-SNPs.

**Table 1:** Prediction of functional ns-SNPs of porcine ANXA5.

SNP ID	Substitution	Polyphen-2		SNAP		SIFT		PhD-SNP	
		Prediction	Score	Prediction	Expected accuracy	Prediction	Score	Prediction	Score
c.165C > A (rs3473888081)	A2E	Benign	0.03	Neutral	0.89	Tolerant	0.11	Neutral	7
c.234G > C (rs711261805)	R25P	Possibly damaging	0.93	Non-neutral	0.58	Deleterious	0.05	Diseased	8
c.286G > T (rs708358082)	L42F	Probably damaging	1.00	Non-neutral	0.58	Deleterious	0	Diseased	8
c.343 T > G (rs81212074)	F61L	Benign	0.00	Neutral	0.60	Deleterious	0.03	Neutral	0
c.404G > A (rs323560041)	V82M	Possibly damaging	0.70	Neutral	0.78	Deleterious	0	Diseased	3
c.754 T > G (rs81212081)	F198L	Benign	0.00	Neutral	0.78	Tolerant	1.00	Neutral	9
c.783G > A (rs705136162)	R208K	Benign	0.00	Neutral	0.92	Tolerant	1.00	Neutral	4
c.830A > T (rs81212083)	T224S	Benign	0.00	Neutral	0.94	Tolerant	1.00	Neutral	8
c.839C > T (rs690134895)	R227W	Probably damaging	0.98	Neutral	0.53	Deleterious	0	Diseased	5

### 3.4 Characterization of the porcine *ANXA5* promoter

**Figure 3:**
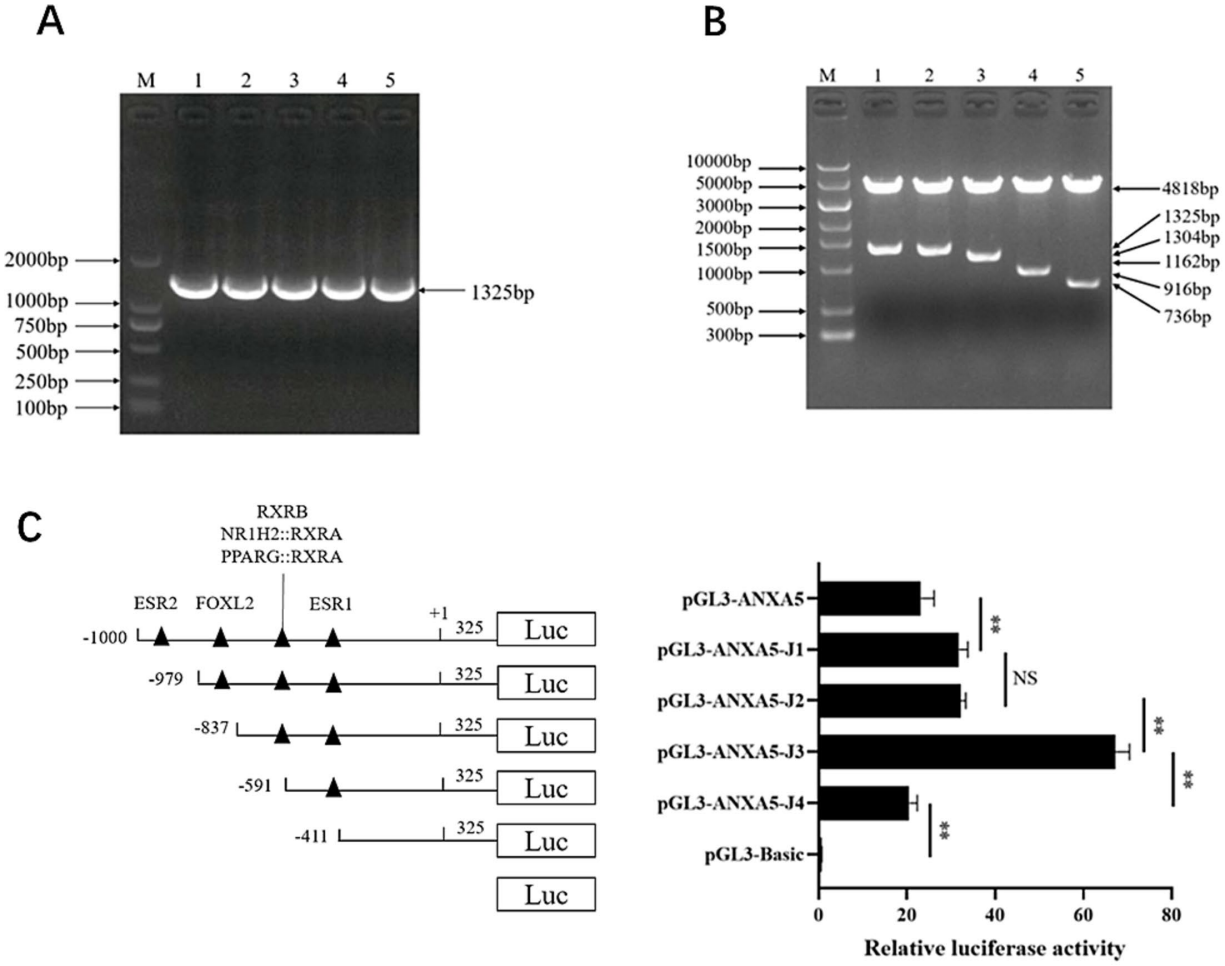
Characterization of ANXA5 promoter. **(A)** Amplification of porcine ANXA5 promoter. **(B)** Verification of pGL3-ANXA5 and 5′-deletion plasmids. **(C)** 5′-deletion analysis of the pGL3-ANXA5. Values were shown as the mean ± SEM (*n* = 3). *p < 0.05; **p < 0.01.

A 1325-bp fragment of porcine *ANXA5* containing a 1,000-bp 5′ flanking region and 325-bp coding sequence was amplified to produce the vector pGL3-ANXA5 (Figure 3A). Bioinformatics analysis revealed the existence of two core promoter regions in this fragment located at −794 to −744 and −702 to −652 bp before the ATG sequence and more than 100 transcription factors were predicted by JASPAR. Based on the literature, four transcription factors (ESR2, FOXL2, RXRB and ESR1) contributing to reproduction were selected. Then, four 5′-deletion fragments targeting the binding sites of these four transcription factors were amplified to construct the corresponding dual luciferase reporter plasmids pGL3-ANXA5-J1 to J4 (Figure 3B).

As shown in Figure 3C, the luciferase activity of pGL3-ANXA5-J1 was significantly higher than that of pGL3-ANXA5 (*p* < 0.01), indicating that the putative ESR2 binding site (−1,000 to −979) repressed the luciferase activity of *ANXA5*. However, there was no difference between pGL3-ANXA5-J1 and pGL3-ANXA5-J2. For the RXRB from −837 to −591 bp and ESR1 from −591 to −411 bp, ANXA5-J3 had significantly greater activity as compared to J2 or J4 (*p* < 0.01), suggesting that RXRB might repress *ANXA5* while ESR1 might be an activation factor.

### 3.5 Association analysis between candidate functional SNPs of porcine *ANXA5* and semen traits

**Figure 4:**
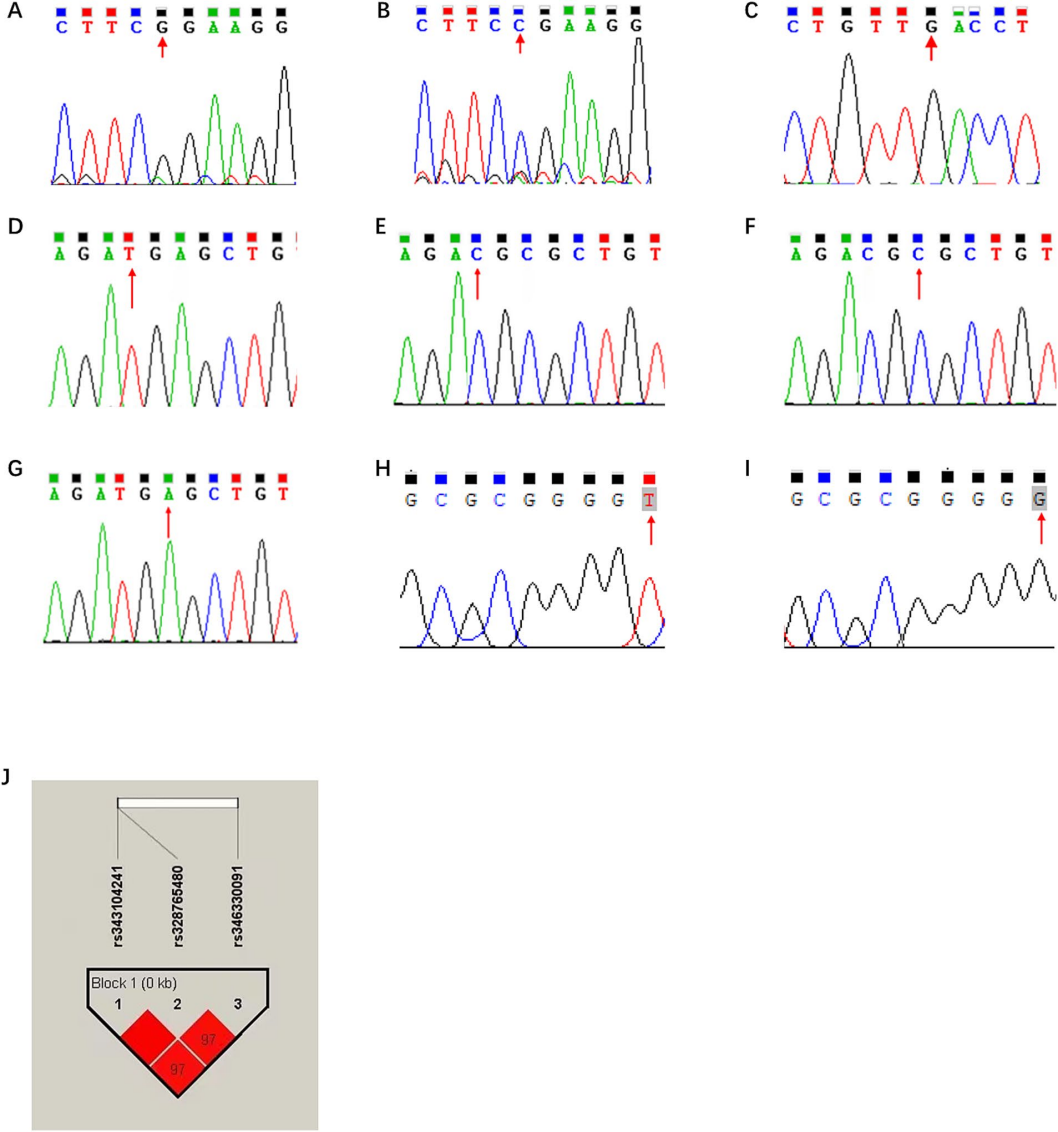
*In silico* analysis of ns-SNPs in porcine ANXA5. **(A)** The sequencing results of rs711261805 locus as G. **(B)** The sequencing results of rs711261805 locus as C. **(C)** The sequencing results of rs708358082 locus with G were analyzed. **(D)** The sequencing results of rs343104241 locus as T. **(E)** The sequencing results of rs343104241 locus as C. **(F)** The sequencing results of rs328765480 locus as C. **(G)** The sequencing results of rs328765480 locus as A. **(H)** The sequencing results of rs346330091 locus as T. **(I)** The sequencing results of rs346330091 locus as G **(J)** Linkage disequilibrium analysis of g.-676 T > C, g.-674C > A and g.-105G > T.

To verify the candidate functional SNPs in the coding sequence, the 549-bp and 319-bp fragments were amplified from 10 Min pigs and 10 Landrace pigs using gene-specific primers (Supplementary Table S1). As shown in Figures 4A,B, the c.234G > C (R25P) polymorphism was validated in the Min pigs, while all 10 Landrace carried G alleles. For the c.286G > T (L42F) polymorphism, all pigs carried the G allele (Figure 4C). Notably, association analysis between these SNPs and Yorkshire boars was not performed.

To explore genetic variations in the regulation region, the *ANXA5* promoter of 10 Min pigs and 10 Landrace pigs was obtained by PCR and 56 SNPs in this region were revealed by DNAMAN. Among them, g.-676 T > C and g.-674C > A were located in the predicted RXRB binding region, and the latter might alter the RXRB binding site, while g.-105G > T was within the ESR1 binding site. According to JASPAR, RXRB would bind to the A allele rather than the C allele, and ESR1 would bind to the T allele but not the G allele at g.-105G > T (Table 2; Figures 4D,I).

**Table 2:** Putative transcriptional factor binding sites in the promoter region of porcine *ANXA5.*

SNP^a^	Variation	Gain^b^	Loss	Prediction database
g.-676 T > C (rs343104241)	T > C	-	-	JASPAR
g.-674C > A (rs328765480)	C > A	NR1H2::RXRA PPARG::RXRA RXRB	-	JASPAR
g.-105G > T (rs346330091)	G > T	ESR1*	-	JASPAR

^a^Based on position before exon 1; ^b,c^Generated after substitution of allele 1 (wild type) with allele 2 (mutant).

For association analysis, the three candidate functional SNPs in the *ANXA5* promoter region of the Yorkshire population were genotyped by DNA direction sequencing. As shown in Table 3, the frequencies of the T allele at g.-676 T > C, A allele at g.-674C > A, and T allele at g.-105G > T were all 0.75. Linkage disequilibrium analysis confirmed that these SNPs were closely linked (Figure 4J). Association analysis indicated that sperm motility was greater for CT animals (g.-676 T > C) as compared to TT individuals (*p* < 0.05). Similarly, sperm motility was greater for the CA or GT genotypes than the AA and TT genotypes (*p* < 0.05) (Table 4).

**Table 3:** Genotype and allelic frequency of three candidate functional SNPs in the promoter of porcine *ANXA5* gene in Yorkshire.

SNP locus	Genotype frequency			Allelic frequency		*χ*^2^ (HWE)
	TC	TT	CC	T	C	0.1
g.-676 T > C (rs343104241)	0.38 (160)	0.56 (232)	0.06 (25)	0.75	0.25	
	AC	AA	CC	A	C	0.1
g.-674C > A (rs328765480)	0.38 (160)	0.56 (232)	0.06 (25)	0.75	0.25	
	TG	TT	GG	T	G	0.07
g.-105G > T (rs346330091)	0.37 (154)	0.57 (236)	0.06 (27)	0.75	0.25	

*χ*^2^ (HWE) chi square test for Hardy–Weinberg equilibrium.

**Table 4:** Association between three candidate functional SNPs in the promoter of porcine *ANXA5* gene and boar semen quality and quantity traits.

Genotypes	Traits	Genotypes (μ ± SE)		
		CC	CT	TT
g.-676 T > C (rs343104241)	Quantity (N)	24	152	219
	VOL (ml)	184.01 ± 9.10	175.60 ± 3.62	180.82 ± 3.01
	SCON (10^8^/ml)	4.29 ± 0.30	4.83 ± 0.12	4.60 ± 0.10
	MOT (%)	90.59 ± 0.57	91.32 ± 0.22 ^a^	90.74 ± 0.19 ^b^
	ASR (%)	8.54 ± 0.84	8.82 ± 0.33	9.28 ± 0.28
		CC	CA	AA
g.-674C > A (rs328765480)	Quantity (N)	24	152	219
	VOL (ml)	184.01 ± 9.10	175.60 ± 3.62	180.82 ± 3.01
	SCON (10^8^/ml)	4.29 ± 0.30	4.83 ± 0.12	4.60 ± 0.10
	MOT (%)	90.59 ± 0.57	91.32 ± 0.22^a^	90.74 ± 0.19^b^
	ASR (%)	8.54 ± 0.84	8.82 ± 0.33	9.28 ± 0.28
		GG	GT	TT
g. -105G > T (rs346330091)	Quantity (N)	26	146	223
	VOL (ml)	180.72 ± 8.76	176.63 ± 3.70	180.36 ± 2.99
	SCON (10^8^/ml)	4.40 ± 0.29	4.80 ± 0.12	4.62 ± 0.10
	MOT (%)	90.62 ± 0.54	91.35 ± 0.23^a^	90.73 ± 0.19^b^
	ASR (%)	8.82 ± 0.81	8.81 ± 0.34	9.25 ± 0.28

Different lowercase letters indicated significant difference (*P* < 0.05).

### 3.6 Effect of g.-105G > T on *ANXA5* transcription

**Figure 5:**
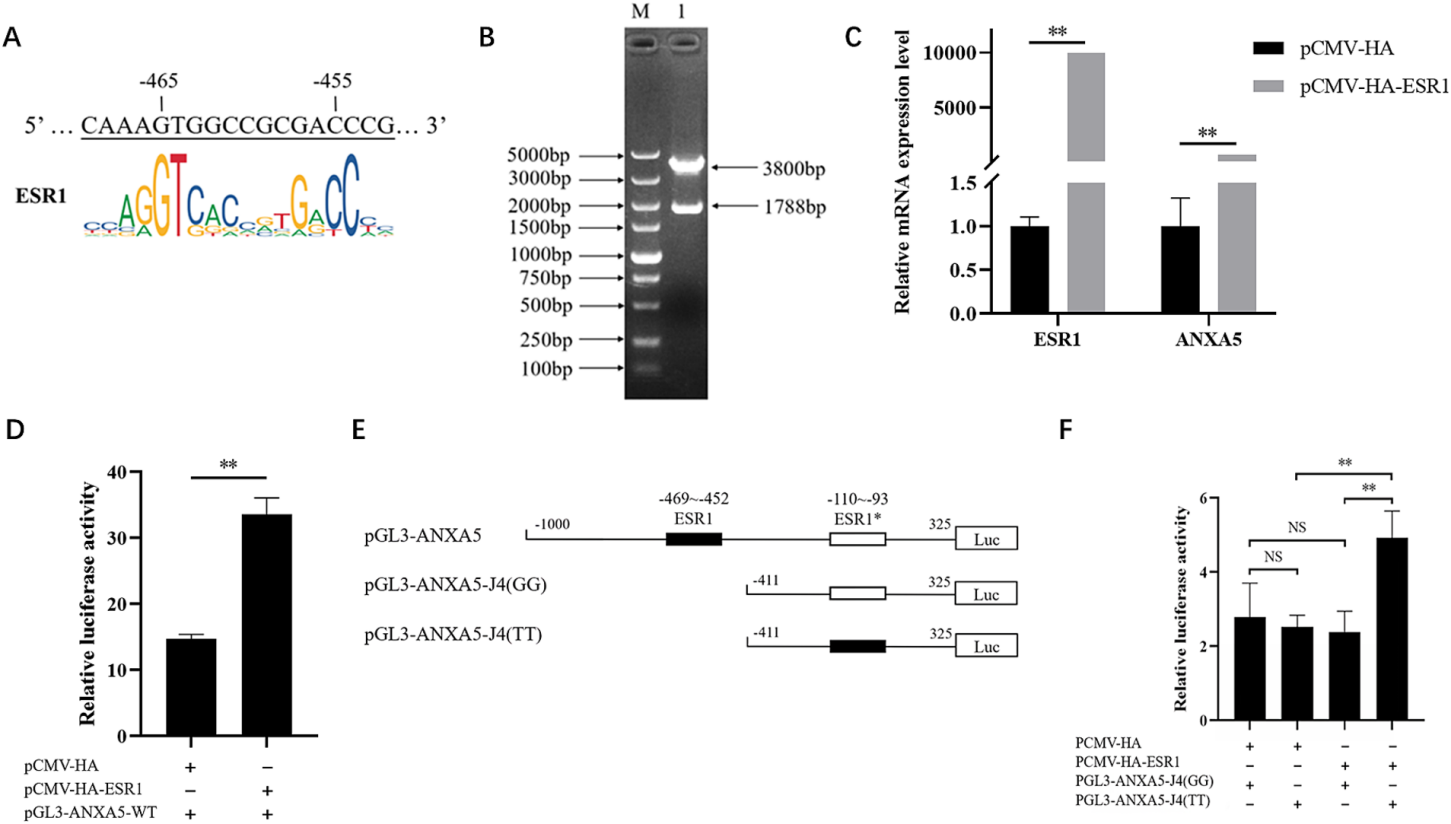
Effect of ESR1 and g.-105G > T on ANXA5 transcription. **(A)** Predictive ESR1 binding site within ANXA5 promoter in ST cells. **(B)** Verification of pCMV-HA-ESR1 plasmids. **(C)** Overexpression of *ESR1* up-regulate ANXA5 mRNA expression. **(D)** Overexpression of *ESR1* up-regulate ANXA5 promoter activity. **(E)** Schematic presentation of putative ESR1-binding sites in ANXA5 promoter. **(F)** Overexpression of *ESR1* up-regulate the promoter activity of ANXA5 with T allele at g.-105G > T. Values were shown as the mean ± SEM (*n* = 3). *p < 0.05; **p < 0.01.

According to the JASPAR results, the predictive ESR1 binding site from −110 to −93 bp existed in the *ANXA5* promoter with allele T at g.-105G > T. To evaluate the effect of this SNP on *ANXA5* mRNA expression, the genomic DNA of ST cells was extracted. The amplified fragment carried the G allele, rather than the T allele, suggesting the ST cells did not carry the putative ESR1 binding site between −110 and −93 bp. Considering the existence of another ESR1 binding site from −469 to −452 bp in the cells (Figure 5A), the effect of ESR1 on *ANXA5* mRNA expression was investigated. As shown in Figures 5B, a 1788-bp coding fragment of *ESR1* was obtained to construct the vector pCMV-HA-ESR1. The results of qRT-PCR analysis showed that overexpression of ESR1 significantly increased *ANXA5* mRNA expression in ST cells (Figure 5C). Luciferase activity was higher in ST cells co-transfected with pCMV-HA-ESR1 and pGL3-ANXA5 than ST cells co-transfected with pCMV-HA and pGL3-ANXA5 (*p* < 0.01; Figure 5D), suggesting an active role of ESR1 in *ANXA5* transcription.

To evaluate the function of g.-105G > T on *ANXA5* transcription, ST cells were co-transfected with the vectors pCMV-HA-ESR1 and pGL3-ANXA5-J4 (GG) carrying the G allele or pGL3-ANXA5-J4 (TT) carrying the T allele (Figure 5E). The results showed that overexpression of ESR1 significantly increased the activity of *ANXA5* with the T allele, but not the G allele (Figure 5F).

## 4 Discussion

In this study, the porcine *ANXA5* gene was first isolated from the testis and RT-PCR analysis showed constitutive expression in all selected tissues, indicating extensive biological functions. The *in silico* analysis revealed that the *ANXA5* was evolutionally conserved among pigs, humans, ruminant, and rodents. Consistently, human and porcine ANXA5 was predicted to interact with S100A12, CASP3, VCL, PLEK, TUBA4A, and TAGLN2. Among these proteins, S100A12 belongs to the S100 family and is reportedly involved in specific calcium-dependent signal transduction pathways and plays roles in inflammation, innate immunity, antimicrobial function, and reproduction (23–25). A recent study found that porcine S100A12 might play a critical role in the establishment and maintenance of pregnancy in pigs (23). Generally, CASP3 is a marker of cell apoptosis and participates in regulation of programmed cell death by cleaving target proteins (26). VCL is a cytoskeletal protein associated with cell–cell and cell-matrix junctions (27), while PLEK, TUBA4A, and TAGLN2 are involved in cell division, proliferation, differentiation, and other processes, suggesting roles of porcine ANXA5 in cell growth.

Previous studies have demonstrated that inhibition of *ANXA5* reduced the proliferation of murine hepatocarcinoma Hca-P cells with about a 25% lymph node metastasis rate (28, 29), human cholangiocarcinoma cells (30), and pre-osteoblastic cells (31). The present study consistently revealed the proliferative effect of ANXA5 on ST cells, which are a collection of immature Sertoli cells (32). Immature Sertoli cells are proliferative, while mature Sertoli cells have lost this capacity. So, the proliferation capacity of immature Sertoli cells causes change to the number of mature Sertoli cells and spermatids that contribute to fertility (33). The proliferation and differentiation of Sertoli cells are also controlled partly by reproductive hormones during different stages of development (34, 35). The effects of *ANXA5* on the development of Sertoli cells, therefore, deserve further exploration.

To explore functional SNPs, the current study focused on the coding region of porcine *ANXA5*. Using the *in silico* method, two functional ns-SNPs were identified, but polymorphisms were not found in the Yorkshire population used in this study, which limited the following association analysis. Considering the notable identification of the causative variation in human *ANXA5*, this study aimed to detect the putative regulatory variants in porcine *ANXA5* and three tightly linked SNPs that were predicted to alter the transcriptional factor binding sites of *RXRB* and *ESR1*. Interestingly, all heterozygous SNPs had better performance, which might be the result of the dominance of alleles or the epistatic effect of non-alleles.

Accumulating evidence suggests that ESR1 is indispensable in male reproduction and fertility. Macheroni et al. (34) reported the role of ESR1 in the proliferation of rat Sertoli cells. The results of the current study consistently revealed the effect of porcine ESR1 on the mRNA expression of porcine *ANXA5.* Nonetheless, future studies are needed to elucidate the relationship between ESR1 and ANXA5 in the development of porcine Sertoli cells.

In humans, it is clear that the H2 haplotype of the *ANXA5* promoter reduced mRNA and protein expression of *ANXA5*, which can increase the risks of placental thrombus and pregnancy loss (36–40). According to the JASPAR results, ESR1 can bind the T allele but not the G allele of porcine ANXA5, and overexpression of ESR1 increased the luciferase activity of the T allele at g.-105G > T, suggesting this SNP might regulate ANXA5 transcription partially by altering the affinity for ESR1. Further studies are needed to determine whether ESR1 regulates ANXA5 transcription via direct promoter binding or indirect mechanisms. Additionally, Gunawan et al. (41) found that g.672C > T in the coding region of *ESR1* was associated with sperm motility and the plasma droplet rate. Future studies are warranted to evaluate the effects of SNPs of *ESR1* and *ANXA5* on semen traits in larger populations with diverse genetic backgrounds to identify novel molecular markers and improve boar semen traits.

In this study, g.-105G > T was identified as a functional variant by regulating ANXA5 transcription, but it remains unclear whether this SNP alters transcription of *ANXA5* through DNA methylation, histone modification, or other transcription factors. Ongoing work will focus on clarifying whether this polymorphism confers epigenetic modification or reactions between transcription factors and contributes to the regulation of *ANXA5*.

## 5 Conclusion

This study demonstrated the proliferative effect of *ANXA5* on immature porcine Sertoli cells. Three SNPs (−676 T > C, -674C > A, and g.-105G > T) were closely linked in the promoter region of ANXA5 and associated with the semen quality of Yorkshire boars. Among them, g.-105G > T has an allele-specific effect on *ANXA5* transcription partially by varying affinity for ESR1. These findings suggest that ANXA5 or g.-105G > T might be a functional candidate gene or mutation for boar semen quality. However, further studies are merited to elucidate the transcriptional regulatory mechanism of ANXA5 during Sertoli cells development.

## Data Availability

The original contributions presented in the study are included in the article/Supplementary material, further inquiries can be directed to the corresponding author.
