# Supplementary material for: Identification of functional SNP associated with sperm quality in porcine ANXA5 that contributes to the growth of immature Sertoli cell
Source: Front Vet Sci. 2025 May 14;12:1576566. doi: 10.3389/fvets.2025.1576566 (PMC12116668; doi:10.3389/fvets.2025.1576566)
Supplement: Supplementary file 1 [file Table_1.docx]

**Table S1.** Primers used for Real-time PCR, SNPs identification and plasmids construction

| **Prime** | **Primer sequence (5’-3’)** | **Annealing**  **temp (℃)** | **Product**  **size (bp)** | **Binding**  **region** |
| --- | --- | --- | --- | --- |
| ANXA5-CDS | F: TATGGTACCATGGCACAGGTTCTCAGAGGCAC | 56 | 966 | - |
|  | R: TCACTCGAGTCAGTCATCTTCGCCTCCACAG |  |  |  |
| ESR1-CDS | F: TTAGAATTCGCATGACCATGACCCTACACACCAA | 58 | 1788 | - |
|  | R: ATTGGTACCTCAGATTGTGGTGGGGAAGTT |  |  |  |
| ANXA5-siRNA | GAGAAAAUCCUGACAGAAATT | - | - | - |
|  | UUUCUGUCAGGAUUUUCUCTT | - | - | - |
| NC | UUCUCCGAACGUGUCACGUTT | - | - | - |
|  | ACGUGACACGUUCGGAGAATT | - | - | - |
| ANXA5-P | F: CGGGGTACCAGGAGGGCACTTTGTCTTA | 55 | 1325 | -1000 |
| ANXA5-J1 | F: TATGGTACCGAGGTCACGGAGGGGAGT | 55 | 1304 | -979 |
| ANXA5-J2 | F: TATGGTACCACGCAGGCGCAGTGAGGC | 55 | 1162 | -837 |
| ANXA5-J3 | F: TATGGTACCGAGGTCACGGAGGGGAGT | 58 | 916 | -591 |
| ANXA5-J4 | F: TATGGTACCACGCAGGCGCAGTGAGGC | 58 | 736 | -411 |
| ANXA5-R | R: CCGCTCGAGGCGATTTTCTGGATTTTGG | - | - | Intron 2 |
| ANXA5-J4(TT) | F: GGGCGCGGGG**T**CTCCGTGGG | - | - | - |
|  | R: CCCACGGAG**A**CCCCGCGCCC | - | - | - |
| ANXA5- Exp | F: TGGATTTGATGAGCGGGCTGATG | 55 | 98 | - |
|  | R: TGCGGGAGGTCAACAGAGTCAG |  |  |  |
| ESR1-Exp | F：TCTGCCAAGGAGACTCGCTACTG | 55 | 83 | - |
|  | R：CAGCCCTCGCAAGACCAAACTC |  |  |  |
| GAPDH-Exp | F: CCCCAACGTGTCGGTTGT | 55 | 83 | - |
|  | R: CCTGCTTCACCACCTTCTTGA |  |  |  |
| PCNA-Exp | F: CTCACCAGCATGTCCAAA | 50 | 90 | - |
|  | R: TAGTGCCAAGGTGTCTGC |  |  |  |
| ANXA5-R25P | F：AATCGGTCCTGCCAATCC | 51 | 549 | - |
|  | R：CACAGCTGGTCACCCTCA |  |  |  |
| ANXA5-L42F | F：GGAAGCAAGATGTAATGG | 51 | 319 | - |
|  | R：AGATAAGTGAGGTGAGCC |  |  |  |
| ANXA5-S | F: AGAAACTGACCCAAGGCAGAAGTT | 63 | 1034 | - |
|  | R: ACACTCCTCGGAAAGCAGAAGGAC |  |  |  |

Note: The underline shows the additional restriction sites: Kpn I (GGTACC), Xho I (CTCGAG) and EcoR I (GAATTC). The bolded bases are the mutated bases.
